# eBird: Engaging Birders in Science and Conservation

**DOI:** 10.1371/journal.pbio.1001220

**Published:** 2011-12-20

**Authors:** Chris Wood, Brian Sullivan, Marshall Iliff, Daniel Fink, Steve Kelling

**Affiliations:** Cornell Lab of Ornithology, Ithaca, New York, United States of America

## Abstract

How do you successfully engage an audience in a citizen-science project? The processes developed by eBird (www.ebird.org), a fast-growing web-based tool that now gathers millions of bird observations per month, offers a model.

Identifying a species is a complex task that relies upon a combination of factors. Observers must be able to process impressions of shape, size, and behavior under variable conditions. As this process takes place, the observer must reconcile these impressions against a list of species most likely to occur at that specific location and date, and constantly recalibrate until the two agree, and the species is correctly identified. Only humans can make this difficult computation of classifying organisms to the species level. And for birds, tens of thousands of people do this every day for fun.

For more than two hundred years the public has contributed significantly to our understanding of bird identification, distribution, and abundance [1]. Building on this tradition, eBird (http://ebird.org/) is a citizen science project that takes advantage of numerous information technologies to engage a global network of birders to report their bird observations to a centralized database [2]. Anyone, anywhere, and at anytime can submit observations of birds via the Internet or through a variety of handheld devices. These amassed observations provide scientists, researchers, and amateur naturalists with data about bird distribution and abundance across varying spatio-temporal extents. All data are free and readily accessible through the Avian Knowledge Network [3],[4]. eBird data have been used in a wide variety of applications, from highlighting the importance of public lands in conservation [5] to studies on evolution [6], and to explore biogeography [7].

eBird is part of the growing field of human computation, which focuses on harnessing human intelligence to solve computational problems that are beyond the scope of existing artificial intelligence algorithms [8]. Many of these services use the web, and include game-based approaches such as the ESP Game, which has collected millions of image labels [9]; FoldIt, which attempts to predict the structure of a protein by taking advantage of humans' puzzle solving abilities [10]; Galaxy Zoo, which has engaged more than 200,000 participants to classify more than 100 million galaxies collected during the Sloan Digital Sky Survey [11]; and reCAPTCHA, which provides security measures on the web while transcribing old print material one word at a time [12]. In this paper, we describe our experiences in developing the eBird network of volunteers, whose observations provide an open data resource containing the most current and comprehensive information on bird distribution, migratory pathways, population trends, and landscape use.

## Know Your Community

eBird uses data collection protocols that match the way birders go birding. The fundamental data gathered with each observation are: species, date, location, and whether all species detected are included on the checklist. Most observations include counts of individuals for each species, and basic information that identifies the observers and describes how the count was conducted (start time, duration, and distance traveled). We chose this relatively simple approach to survey design in order to engage the largest number of participants, as increasing complexity of protocols in citizen science projects tends to decrease the number of participants in those projects [13]. While this basic protocol captures the majority of eBird observations, birders also have the option to provide additional detailed information for each observation, such as age and sex, breeding behavior, and additional comments.

A significant aspect of the data that birders contribute to eBird is that each observation has an exact date and is linked to a point on the map. This provides the opportunity to link eBird data with a variety of covariate data that potentially influence bird occurrence, such as weather, climate, habitat, and human population density. This allows the eBird community to focus on what it does best: finding, identifying, and counting birds.

While we encourage all eBird contributors to conform to the protocol standards we have developed, we also allow users to enter data in a variety of other ways, even though these observations may be of reduced analytical value. For example, while eBird strives to gather detailed location and temporal specificity, historic data often can only be entered at the county or even state level. Other counts may be entered that cover long distances, or that simply report incidental or random observations of birds. Flexible data entry increases initial involvement, and once involved with eBird, we can transition users toward improved data collection techniques. We have found that by providing various incentives and training on the eBird web site, we can convert most of these initially casual observers into higher quality data collectors who use the effort-based eBird protocols (see below).

## Maximizing Participation

Initial reaction to eBird from the birding community was lukewarm, and there was little growth in participation. Recognizing this, we modified our development approach to focus on building tools that appeal to, and provide a service for, birders. By building tools that allowed birdwatchers to keep track of their observations, view their personal bird lists, and compare their observations with others, we built upon the established norms of the birding community. The result was rapid and sustained growth. For example, over the past six years, eBird's usage has increased dramatically, with the number of checklist submissions to exceed 1.7 million from more than 210 countries in 2011.

eBird participation generally follows Pareto's Law (the 80/20 law), with the majority of the data submitted by a very active subset of “power users”. For example, 90% of checklists submitted to eBird have come from the most active 10% of users. Understanding the eBird power users allowed us to develop eBird features focused on these high-level users, in the belief that this would in turn increase broader participation. Over time, this model has been successful in greatly increasing participation. Having the majority of data submitted by committed, repeat users helps maintains high data quality, since these users show a commitment to the project, and an understanding of its best practices, as well as a clear investment in the community. One should not confuse eBird power users with “expert birders”. While many eBird power users are indeed experts in terms of field skills, detecting, and identification, many other birders with more modest skills are also power users who may only enter data from their backyard or local area. They may not be able to detect very rare species in their region on the basis of sound alone, but they are proficient with a subset of regularly occurring birds, and they provide eBird with repeated samples of the birdlife in a defined area over time. Moreover, through sustained participation they develop a higher level of eBird expertise.

## Changing Behavior through Tools and Visualizations

Once we are able to engage a birder in eBird, we focus on modifying behavior so that the checklists entered are more useful for research and conservation. We rely upon two techniques to encourage higher quality submissions: education about how and why making small changes to the way you go birding can improve the value of your data; and incentives that reward participants for collecting data following more rigorous protocols.

**Figure 1:**
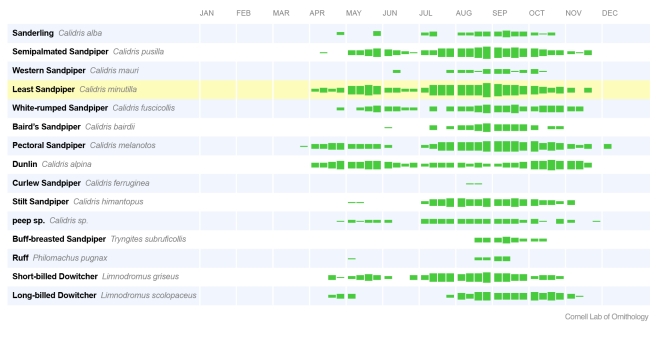
eBird frequency bar chart for Montezuma National Wildlife Refuge. Data output tools such as the eBird bar charts serve a variety of inter-related purposes from engaging users, to encouraging better data collection, to informing land management decisions. These bar charts for Montezuma National Wildlife Refuge (http://ebird.org/ebird/GuideMe?step=saveChoices&getLocations=ibas&parentState=US-NY&bMonth=01&bYear=1900&eMonth=12&eYear=2011&reportType=location&ibas=US-NY_MOWC&continue.x=34&continue.y=13&continue=Continue) help birders plan trips to see shorebirds, and encourage birders to enter complete checklists of all species to ensure their data are used in output like this. They also provide refuge managers with details on the seasonal occurrence of shorebirds to inform management actions, such as raising or lowering water levels to create feeding habitat for birds. Much of the simple output in eBird uses “frequency of detection”, i.e., the percentage of checklists that report a species. By clicking on the name of any species, like Stilt Sandpiper (http://ebird.org/ebird/GuideMe?cmd=decisionPage&speciesCodes=stisan&getLocations=states&states=US-NY&bYear=1900&eYear=2011&bMonth=1&eMonth=12&reportType=species&parentState=US-NY), you can interact with the data to view locations where the species was found, explore high counts, and view birds per hour. Image credit: Cornell Lab of Ornithology.

Users receive educational training regarding the scientific reasons for certain methodologies in eBird, and learn improved data collection techniques from the eBird project leaders and volunteers. Even more effective in transforming behavior are incentives in the form of visualizations and data output tools that demonstrate why better data collection techniques benefit our users on a personal level. We developed the eBird bar charts (Figure 1) to tackle one of our biggest challenges: encouraging birders to move from reporting two or three highlight species seen during a day of birding, to submitting complete checklists of all species seen and heard from a series of more refined stops throughout the day. Bar charts are intuitive data visualizations that birders have traditionally used to understand the seasonal timing of birds within a region. Popular bird-finding guides often include these charts to give a visual representation of when birds are expected to be present in a region (usually a state). eBird has automated the presentation of bar charts and similar visualizations that depict frequency of occurrence. Immediately, birders could understand the seasonal patterns of bird movements within any region of interest. The key is that in order for these visualizations to work best, observers must submit *complete lists of all species recorded*. Birdwatchers now have a personal incentive to improve their data collection techniques. Equally important, they now have a reason to regularly enter records that help develop these bar charts. We also provide simple tabular outputs (eBird's “Top 100”) that give recognition to the users who have submitted the most checklists or observed the largest number of species. This popular and important tool helps maintain high levels of participation by allowing our community to engage with each other in competitions to submit more complete checklists or see more species. This combination of educational features and data output tools that create personal reward are very powerful motivators for generating increased data volume and quality.

## Ensuring Data Quality

High data quality is critical for achieving scientific goals and for engaging users. Our approach to data quality is to develop tools that allow experts to develop regional filters that identify outlier records. Editors establish a maximum number of individuals that may be entered for every species and each month for a given region. These outliers are the same kind of records that amateur and professional ornithologists have focused on in keeping regional records of bird occurrence. The tools in eBird provide an easy way for our regional editors to isolate and follow-up on unusual records with the original observers. These volunteers provide an enormous service to eBird, as their expertise greatly improves the quality of eBird data. To date, our network of over 450 regional experts has reviewed more than 3.5 million records.

## Adding Value with Statistical Modeling

**Figure 2:**
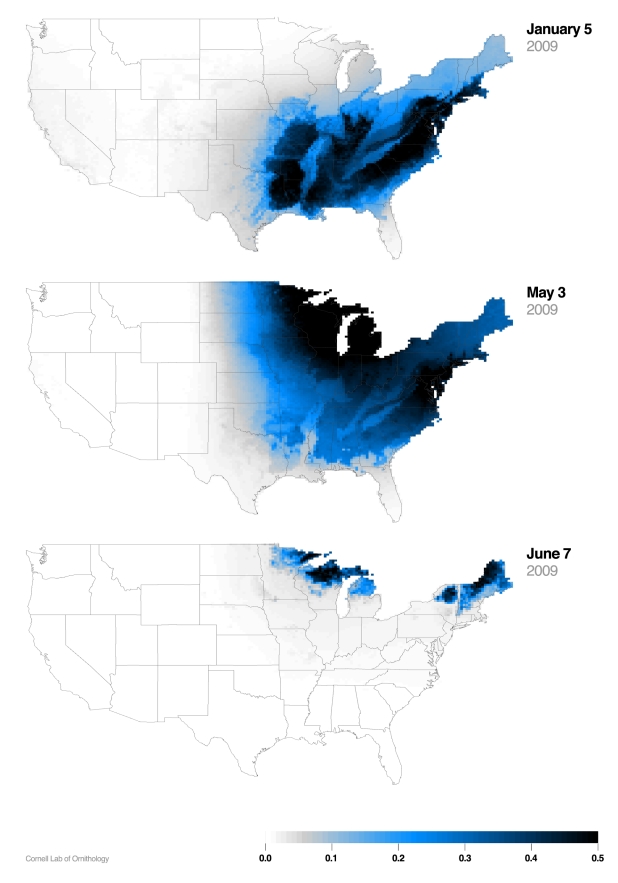
White-throated Sparrow distribution. Figure 2 illustrates a STEM distribution estimate for White-throated Sparrow (*Zonotrichia albicollis*), a migratory songbird that winters in the southeastern US and breeds in the northeastern US and eastern Canada. This occurrence map shows the probability of encountering the species (maximum 50% probability) on a 1-hour birding walk starting at 7:00 a.m. for 5 January, 3 May, and 7 June 2009. Using a temporal sequence of daily continental-scale distribution estimates allows quick assessment of such things as the rate of arrival and departure from the wintering grounds, migratory corridors, and regions of particular importance for breeding, wintering, or migratory stopover. Over time, these same models will help identify and quantify changes. Image credit: Cornell Lab of Ornithology. To view an animation of White-throated Sparrow distribution throughout the year, visit http://ebird.org/content/ebird/about/occurrence-maps/white-throated-sparrow.

Despite the growing amount of species occurrence data collected by eBird, species observations are, at best, sparsely distributed in space and through time. This has motivated us to develop spatially and temporally explicit models of species occurrence by relating environmental features that are important to a species (e.g., habitat, climate, elevation) to observational data. Once related, statistical models can make predictions at unsampled locations and times. To facilitate this process, eBird observations have been linked through observation location and time to a large number of local environmental descriptors, such as remotely sensed habitat information from the National Land Cover Database, and vegetation phenology from MODIS [3]. We use this data to estimate species distributions with the SpatioTemporal Exploratory Model (STEM), designed to utilize both the broad extent and fine resolution information collected by eBird [14]. STEM has been used to estimate year-round distributions for over 100 terrestrial bird species with a wide variety of distinct migration pathways and local habitat associations (Figure 2) [5]. With this information, ecologists will be better able to identify, prioritize, and coordinate conservation actions across broad landscapes.

## Building a Global Network

Many birds migrate throughout much of the Western Hemisphere and beyond, and always without regard to geopolitical boundaries. While the Lab's research focus is the Western Hemisphere, our audience actively encouraged us to expand eBird globally, thereby allowing them to keep track of their bird observations from anywhere around the planet. To accomplish this, we had to develop a broad and continually growing network of global partnerships.

The goal of gathering observations of birds to further science and conservation is shared by hundreds of organizations that have local and regional expertise. By partnering with these groups, we could further our mutual goals. For example, the Mexican federal Comisión Nacional para el Conocimiento y Uso de la Biodiversidad (CONABIO) was interested in gathering observations of birds in Mexico. Together, we created a version of eBird translated to Spanish for Mexico called aVerAves. All data from this portal go into eBird and vice versa—the two share the same database, but CONABIO is able to develop news and features directly relevant to a Mexican audience. CONABIO is able to encourage birders to enter data through local promotion and engagement, and ultimately build a community that understands and values their work.

Building upon this model partnership in Mexico, we continue to develop collaborations around the world. These are tailored to the individual needs of a specific country or region. For instance, in the United Arab Emirates, we have focused on developing data quality filters and uploading over 195,000 records from existing databases—eBird provided a better way to visualize, store, and access these records than previously existed. Other collaborations have led to the development of specific research projects ranging from grassland bird monitoring in the Chicago Wilderness (Bird Conservation Network eBird), to winter and summer bird atlases. For example, our partner in Chile, the Red de Observadores de Aves y Vida Silvestre de Chile (ROC), focuses on developing eBird as a tool for Chilean birdwatchers and researchers. In doing so, they have made eBird the primary data repository for all bird data in Chile, including official bird surveys conducted by the Coporacion Nacional Forestal (CONAF). We worked closely with the ROC to bring breeding behavior codes into eBird, which has allowed them to initiate the first countrywide breeding bird atlas for a South American country. In building this tool focused on Chilean goals, we were able to deploy this functionality for the entire world. The flexibility of eBird provides a database as well as a forum for engaging users that local researchers and conservationists can develop to achieve local goals.

## Conclusion

Identifying ecological patterns across broad spatial and temporal extents requires novel approaches and methods for acquiring, integrating, and analyzing diverse environmental observational data. Engaging volunteers to collect the required data across such broad scales has tremendous potential to advance scientific understanding in ecology, and improve species conservation and management. Here we have described eBird, a global network of volunteers that collects massive quantities of species occurrence data in one of the largest citizen science projects in existence.

eBird has found a balance between data quantity and quality that has allowed us to gather a sufficient volume of useful data to provide a resource for the study of bird populations at spatial and temporal scales heretofore unattainable. Our experience has shown that an uncomplicated protocol and appropriate rewards for volunteer participation are very important for the recruitment and retention of large numbers of volunteers. Even after the data are collected and passed through a quality control process, we have found that existing methods for analysis still may not always be suitable for use with such broad-scale observational data [15]. It is only through an ongoing collaboration between ecologists, statisticians, and computer scientists that the development of novel methods for extracting biological insights from these data has been enabled.

eBird currently provides a resource on bird occurrence that has allowed us to extend our study of patterns of occurrence of many North American species throughout a bird's entire life history. As the growth in participation in eBird continues, we will be able to extend these analyses globally, and in so doing enable land managers and conservation biologists to better coordinate national and international conservation efforts with the aid of citizen science data.

## References

[1] Barrow M. V (1998). A passion for birds: American ornithology after Audubon.

[2] Sullivan B. L, Wood C. L, Iliff M. J, Bonney R. E, Fink D (2009). eBird: a citizen-based bird observation network in the biological sciences.. Biological Conservation.

[3] Munson M. A, Webb K, Sheldon D, Fink D, Hochachka W. M (2009). The eBird reference dataset.. http://www.avianknowledge.net/content/features/archive/eBird_Ref.

[4] Iliff M, Salas L, Inzunza E. R, Ballard G, Lepage D, Rich T (2009). The Avian Knowledge Network: a partnership to organize, analyze, and visualize bird observation data for education, conservation, research, and land management..

[5] NABCI U. S (2011). The state of the birds 2011 report on public lands and waters.

[6] McCormack J. E, Zellmer A. J, Knowles L. L (2009). Does niche divergence accompany allopatric divergence in Aphelocoma jays as predicted under ecological speciation?: insights from tests with niche models.. Evolution.

[7] Klicka J, Spellman G. M, Winker K, Chua V, Smith B. T (2011). A phylogeographic and population genetic analysis of a widespread, sedentary North American bird: the hairy woodpecker (Picoides villosus).. The Auk.

[8] Law E, Ahn L. v (2011). Human computation. Synthesis lectures on artificial intelligence and machine learning.

[9] Ahn L. v, Dabbish L (2004). Labeling images with a computer game. Proceedings of the SIGCHI Conference on Human Factors in computing Systems.

[10] Popovic Z, Cooper S, Khatib F, Treuille A, Barbero J (2010). Predicting protein structures with a multiplayer online game.. Nature.

[11] Raddick M. J, Bracey G, Gay P. L, Lintott C. J, Murray P (2010). Galaxy Zoo: exploring the motivations of citizen science volunteers.. Astronomy Education Review.

[12] von Ahn L, Blum M, Langford J (2004). Telling humans and computers apart automatically.. Communications of the ACM.

[13] Bonney R, Cooper C, Dickinson J, Kelling S, Phillips T (2009). Citizen science: a developing tool for expanding science knowledge and scientific literacy.. BioScience.

[14] Fink D, Hochachka W. M, Zuckerberg B, Winkler D. W, Shaby B (2010). Spatiotemporal exploratory models for broad-scale survey data.. Ecological Applications.

[15] Hochachka W. M, Caruana R, Fink D, Munson A, Riedewald M (2007). Data-mining discovery of pattern and process in ecological systems.. Journal of Wildlife Management.

